# The Perioperative Use of Codeine and Tramadol in Pediatric Population

**DOI:** 10.31480/2330-4871/198

**Published:** 2025-02-10

**Authors:** Wiriya Maisat, Koichi Yuki

**Affiliations:** 1 Department of Anesthesiology, Faculty of Medicine Siriraj Hospital, Mahidol University, Bangkok, Thailand; 2 Department of Anesthesiology, Critical Care and Pain Medicine, Boston Children’s Hospital, USA; 3 Department of Anaesthesia and Immunology, Harvard Medical School, USA

**Keywords:** Codeine, Tramadol, CYP2D6

## Abstract

Codeine and tramadol are two commonly used opioids for pediatric pain. However, death and severe respiratory distress cases following codeine administration illustrated there would be a significant heterogenous response to these opioids depending on CYP2D6 phenotypes, leading to FDA warning against pediatric use. Here we will comment on the current status of pediatric pain management in the context of codeine and tramadol.

## Introduction

Codeine had been the most popular analgesics prescribed to children after tonsillectomy and/or adenoidectomy for many years [1]. However, the FDA identified total of 13 adverse events in children associated with codeine dated from 1969 to 2012 in its Adverse Event Reporting system [2]. 10 patients died and 3 patients had severe respiratory depression due to “overdose”. 8 cases involved adenotonsillectomy and 3 cases of codeine use were in the setting of respiratory infection. In most of these cases, the children seemed to receive appropriate doses of codeine. In some cases, children had genetic polymorphisms, thus high functioning converting enzyme from codeine to morphine [3–6]. The information triggered the FDA to release a black box warning against codeine in 2012. However, the follow-up study in 2015 suggested that 1 out of 20 pediatric patients was still prescribed codeine after adeno-tonsillectomy [7].

In 2017, the FDA implemented several labeling changes to further restrict the use of codeine and tramadol in pediatric patients: 1) *Contraindication* against using codeine for pain or cough and tramadol for pain in children under 12 years; 2) *Contraindication* against using tramadol for pain in children under 18 years after tonsillectomy or adenoidectomy; 3) *Warning* against using these drugs in adolescents (12–18 years) with obesity, obstructive sleep apnea, or severe lung disease due to increased respiratory risk; and 4) *Strengthened warning* advising against breastfeeding while taking codeine or tramadol, as opioid metabolism variability may cause severe neonatal respiratory depression or death [8]. Here we will further follow up the status of codeine and tramadol use in pediatric cohort.

## The Pharmacology of Codeine and Tramadol

Codeine is a prodrug and metabolized via three major pathways. More than 70% of codeine is metabolized by UDP-glucuronosyltransferase-2B7 (UGT2B7) into active codeine-6-glucuronide, 10% is metabolized by the cytochrome P450 (CYP) CYP2D6 into another active metabolite, morphine, and 5–10% is metabolized by CYP3A4. Codeine has been approved for pain control and used off-label for cough management in the US. Tramadol is a synthetic analog of codeine and metabolized via two major pathways. It is metabolized by CYP2D6 into its active metabolite, O-desmethyltramadol, and metabolized by CYP3A4 into inactive N-desmethyltramadol. Both morphine and O-desmethyltramadol have roughly 200-fold higher affinity to the μ-opioid receptor compared to their parent compounds [9].

Hydrocodone and oxycodone, while they are not included in the FDA warning in 2017, are other opioids that depend on CYP2D6 to convert them to their more potent compounds. They are converted to hydromorphone and oxymorphone, respectively. Hydromorphone and oxymorphone have roughly 10~30-fold and 40-fold higher affinity to the μ-opioid receptor compared to their parent compounds, respectively [9].

## Variation in CYP2D6 Enzymatic Activity

Variations in CYP2D6 alleles have been studied. CYP2D6*1 is a wild-type with normal enzymatic activity. But there are many variants with highly variable enzymatic activity [10].

About 5–10% of people are consider poor metabolizers as they have no functional CYP2D6 alleles, thereby its enzymatic activity. In contrast, 1~2% are ultra-rapid metabolizers with multiple copies of functional CYP2D6 alleles and increased enzymatic activity.

## Follow-Up after the FDA Warning in 2017 and Future Considerations

While the 2017 FDA warning did not completely omit prescription to pediatric population, a significant decline of codeine and tramadol usage was observed. Similarly, a significant decline in the use of tramadol was observed [11]. Similar restriction was instituted to other countries with a significant reduction in their usage [12]. While significant respiratory depression and subsequent death is a major issue, poor metabolizers may experience lack of responses. Codeine-6-glucuronide has analgesic potency [13], but poor metabolizers have less analgesic efficacy from codeine [14].

However, about 75% of children have normal CYP2D6 enzymatic function [15]. The Clinical Pharmacogenetics Implementation Consortium proposed some guidelines to use codeine based on CYP2D6 phenotype. In fact, CYP2D6 genotyping have been performed at multiple institutions as part of clinical practice [16]. This in fact could potentially help how we would treat pain for individual patients. Does this have any perioperative implication? This approach was examined in adult patients receiving total joint arthroplasty with successful execution [17]. For this study, CYP2D6 phenotype-driven pain management arm received CYP2D6 phenotyping using their buccal samples preoperatively. Once the result came back, a clinical pharmacist provided a standardized consult note to communicate prescription recommendations based on CYP2D6 phenotype. Poor metabolizers, intermediate metabolizers and ultra-rapid metabolizers were recommended to avoid codeine, tramadol, hydrocodone and oxycodone and use to an alternative opioid (e.g. morphine, hydromorphone) or non-opioids (such as NSAID). For normal metabolizers, tramadol was recommended as the preferred opioid as its lower risk of misuse [18,19]. In the in-patient setting, opioids are used under significant healthcare provider observation for their effectiveness and safety. However, with an increased number of day surgery and early postoperative discharge cases, postoperative pain management has been done more outside hospital settings. Thus, adequate pain management without significant risks is critical for successful healthcare delivery. With still relatively limitation of outpatient short-acting opioid selection, this kind of approach would possibly create a better perioperative management of patients.

## Challenges in Pediatric Pain Management

The strict use of tramadol and codeine in pediatric populations comes with significant challenges in some countries. In many settings outside the US where access to stronger opioids like oxycodone is limited, tramadol and codeine are sometimes used for non-cancer pain in children when acetaminophen is not enough and stronger opioids like morphine are unnecessary.

For children with chronic pain, morphine syrup may sometimes be required for effective pain relief, but its use introduces additional social and logistical difficulties. For instance, administering morphine during school hours may raise concerns among teachers or school staff about potential side effects, such as sedation or respiratory depression. There is also a risk of the medication being misplaced, stolen, or accidentally taken by other children, leading to serious complications. Additionally, there is often a perception among school staff that a child receiving morphine is severely ill and should no longer attend school, resulting in recommendations for termination of the child’s schooling. In contrast, weak opioids such as tramadol or codeine are often more acceptable in the school environment. Staff in the school’s nursing room are usually more comfortable handling these medications, storing, and administering them to child when needed. These factors made tramadol and codeine more practical options for managing pain in children in non-hospital settings.

Given the practical challenges of pediatric pain management, decisions around tramadol or codeine often emphasize practicality and affordability rather than pharmacogenetic factors. From safety standpoint, they should not be used in patients who show hyper-sensitivity to codeine and tramadol (i.e. ultrametabolizers). While understanding these CYP450 enzyme variations is important for assessing drug metabolism and risks, these considerations might have been secondary in clinical practice. Many clinicians prescribe these medications while monitoring their effects closely, even in cases where regulatory guidelines advise against their use. This pragmatic approach reflects the need to balance effective pain relief with the limitations of real-world healthcare system, ensuring that children receive adequate care despite these challenges.

In conclusion, codeine and tramadol had been very favorable drugs of choice in pediatric pain management for a long time. With a significant heterogeneity of codeine and tramadol responses based on CPY2D6 phenotype, pharmacogenetics approach provides some solution in resourceful healthcare setting. However, in countries where resources are rather limited, other reasonable approach may need to be developed for pediatric pain management as opioid selection is rather limited.
